# Assessing the clinical applicability of large language models for microwave ablation guidance

**DOI:** 10.1016/j.ejro.2026.100804

**Published:** 2026-08-03

**Authors:** René Michael Mathy, Fabian Schmitz, Hans-Ulrich Kauczor, Hyungseok Jang, Sam Sedaghat

**Affiliations:** a Department of Diagnostic and Interventional Radiology, University Hospital Heidelberg, Heidelberg, Germany; b Department of Radiology, University of California Davis, Sacramento, USA

**Keywords:** Microwave ablation, Large language models, ChatGPT, Artificial intelligence, Interventional radiology, Clinical decision support

## Abstract

**Purpose:**

To evaluate the performance of five language models (LLMs)—ChatGPT, Gemini, Meta, Claude, and DeepSeek—in providing clinically relevant advice on microwave ablation (MWA), a minimally invasive therapy for tumor treatment.

**Methods:**

A total of 27 standardized questions covering seven thematic domains of MWA were presented to each LLM using a single-input approach. Responses were independently evaluated by three senior radiologists using three customized 4-point rating scales: Overall Quality Score (OQS), Understandability Score (US), and Implementability Score (IS). The mean of these metrics was calculated as the Mean Quality Score (MQS). Interrater reliability was assessed using intraclass correlation coefficients (ICC).

**Results:**

540 ratings were performed. All LLMs successfully generated relevant responses. ChatGPT achieved the highest MQS (3.65 ± 0.54), outperforming Claude (3.47 ± 0.59) and DeepSeek (3.47 ± 0.60), while Gemini (3.00 ± 0.55) and Meta (2.95 ± 0.61) scored significantly lower (p < 0.05). ChatGPT also ranked highest across all subcategories, particularly in Effectiveness and Outcomes (3.73 ± 0.44), Risks and Complications (3.89 ± 0.32), and Post-Procedure Care (3.89 ± 0.32). Interrater reliability was moderate to good (ICC = 0.56–0.75), confirming consistent evaluation.

**Conclusion:**

All examined LLMs demonstrated the ability to provide meaningful advice on MWA. However, ChatGPT showed the best overall accuracy, clarity, and clinical applicability. Claude and DeepSeek achieved comparable results in specific areas. While these findings highlight the promising potential of LLMs in supporting clinical decision-making, human oversight and domain-specific validation remain essential.

## 1 Introduction

Microwave ablation (MWA) has emerged as a pivotal modality in minimally invasive therapies, especially for treating tumors in the liver, lungs, kidneys, and other organs [1]. By using electromagnetic waves to generate localized heat, MWA achieves effective tissue destruction with advantages such as reduced procedure times, broader ablation zones, and compatibility with imaging modalities like ultrasound or CT [2]. However, the efficacy and safety of MWA are significantly influenced by clinical decision-making, including proper patient selection, parameter optimization, procedural execution and post-procedure follow-up [3], [4], [5], [6], [7], [8]. Providing accurate, timely, and context-specific advice is thus essential for ensuring optimal outcomes in MWA procedures. Since hospitals worldwide are confronted with escalating demands and limited resources due to demographic changes and medical progress [9], [10], [11], automated help to support specialized physicians in giving advice on medical procedures to referring physicians as well as patients would be highly welcome.

In recent years, large language models (LLMs) have demonstrated substantial potential as tools for knowledge retrieval, decision support, and guidance in various medical domains [12], [13], [14]. Models such as GPT, Claude, Gemini, Meta and DeepSeek have been trained on vast corpora of text, enabling them to provide human-like responses to a wide array of queries. Although initial investigations into the use of LLMs in the medical field have already been carried out [15], [16], [17], their application in specialized fields like MWA, which require domain-specific expertise and nuanced understanding, remains underexplored [13]. This raises critical questions: How effective are LLMs in offering practical and accurate advice for MWA procedures? Do they exhibit biases or limitations that could affect clinical recommendations? How do different LLMs compare in terms of accuracy, comprehensiveness, and usability for MWA-related queries. Do they give wrong and thus potential harmful advice such as recommending ablations despite clear contraindications, e.g. an ablation near vital structures like the central bile ducts?

This paper aims to address these questions by systematically evaluating the performance of various LLMs in the context of providing advice for MWA. By benchmarking responses according to a novel scoring system specifically designed for evaluating LLMs [16], [18], we seek to illuminate the strengths and weaknesses of these models in supporting healthcare professionals.

## 2 Materials and methods

### 2.1 Study design and question development

This study presents a comparative analysis of five state-of-the-art LLMs to evaluate their performance in providing advice related to MWA. The selected models included ChatGPT−4o (OpenAI, USA; abbreviated as ChatGPT), Gemini Advanced (Google, USA; abbreviated as Gemini), Meta AI based on Llama 3.1 405B (Meta AI, USA; abbreviated as Meta), Claude 3.5 Sonnet (Anthropic, USA; abbreviated as Claude), and DeepSeek (China). Each LLM was presented with an identical set of 27 standardized questions designed to assess their capabilities in addressing clinically relevant aspects of MWA. These questions were written from a professional clinical perspective, e.g. a referring physician, who is not familiar with this procedure only performed in specialised centres.

The development of these questions followed a structured, multi-source approach. Items were derived from current literature, international guidelines, and targeted LLM and Google queries, ensuring comprehensive coverage of MWA-related topics. The initial draft was reviewed and refined by three board-certified radiologists, each with clinical experience in ablation techniques and without the help of a LLM in this final step. Their expertise ensured the clinical relevance, accuracy, and thematic coherence of the questions.

A consensus-driven process was used to finalize and categorize the questions into seven key thematic domains, each representing a critical area of clinical relevance in MWA:

1. Indications and Suitability
2. Procedure Details
3. Effectiveness and Outcomes
4. Risks and Complications
5. Post-Procedure Care
6. Patient Selection and Preparation
7. Technical Aspects

Each domain contained three to four targeted questions, resulting in a total of 27 comprehensive prompts (Table 1). All model interactions were conducted between within a time frame of 10 days and the latest available versions of each LLM were used. Queries were administered in English using an identical, single-turn input format without additional system instructions or few-shot prompting to minimize bias. To reflect real-world use, a first-input approach was applied—each model received the prompt once, without iterative refinement. All five LLMs were accessed through their respective official web-based interfaces. To reflect the experience of a standard clinical user, all interactions were conducted using default 'standard' settings; specifically, no custom system instructions were provided, and parameters such as 'temperature' or 'top-p′ were kept at their factory-default values. Web-browsing, live-grounding, and citation features were left in their default states and none of the models exhibited safety-filter triggers that prevented responses to the clinical microwave ablation queries. All models were operated within their standard context window constraints, which were sufficient for the single-turn, text-based queries used in this study. We tried to simulate a real-world scenario. Therefore, we avoided previous optimization and used standard settings.

**Table 1:** All questions that were presented to the LLMs, sorted by categories.

**Indications and Suitability**
What are the primary indications for microwave ablation?
What types of tumors are most effectively treated with microwave ablation?
What tumor locations are most effectively treated with microwave?
What are the size and number limitations of lesions that can be treated with microwave ablation?
**Procedure Details**
How is the microwave ablation procedure performed?
What imaging techniques are used to guide the procedure?
How long does the procedure typically take?
Is the procedure performed on an outpatient basis or does it require hospitalization?
What type of anesthesia or sedation is used during the procedure?
**Effectiveness and Outcomes**
What are the success rates and outcomes associated with microwave ablation?
What is the risk of rest tumor or local recurrence after microwave ablation?
How does microwave ablation compare to other treatment options like surgery or radiofrequency ablation?
What are the long-term outcomes for patients undergoing microwave ablation?
What are factors influencing short- and long-term outcomes for patients undergoing microwave ablation?
**Risks and Complications**
What are the potential risks and complications of microwave ablation?
How often do these complications occur, and what are the most common ones?
What measures are taken to prevent these complications?
How are these complications managed if they arise during or after the procedure?
**Post-Procedure Care**
What is the typical recovery time after microwave ablation?
What kind of follow-up care and monitoring are required post-procedure?
What signs and symptoms should prompt immediate medical attention after the procedure?
**Patient Selection and Preparation**
What pre-procedure evaluations are necessary for a patient to be considered for microwave ablation?
What are absolute and relative contraindications for microwave ablation?
What instructions should be given to the patient in preparation for the procedure?
**Technical Aspects**
What equipment is used for microwave ablation?
How does microwave ablation work?
How is microwave ablation different from other treatments like radiofrequency ablation or cryoablation?
What are typical technical limitations or challenges associated with the procedure?

### 2.2 Evaluation by radiologists

Responses from the five LLMs were independently assessed by three senior radiologists experienced in MWA. They used a recently established quality scoring system [16], [18], [19] consisting of the following three scoring attributes to evaluate each response:

1. Overall Quality Score (OQS) – accuracy, completeness, and relevance
2. Understandability Score (US) – clarity and readability
3. Implementability Score (IS) – practicality and clinical applicability

Each criterion was rated on a 4-point scale (1 = insufficient, 4 = very good). The mean of these three metrics was calculated as the Mean Quality Score (MQS).

A total of 540 ratings were generated (27 questions × 5 models × 3 raters). Inter-rater consensus was reached via discussion meeting and re-scoring afterward in cases of disagreement.

During the evaluation phase, the three senior radiologists were not formally blinded to the identity of the LLMs. This decision was based on the fact that different models possess distinct linguistic 'fingerprints'—such as specific formatting preferences, typical sentence structures, and introductory phrases—that make complete anonymization ineffective for expert raters. To ensure an objective assessment, however, all model responses were standardized into a uniform plain-text format before being presented to the evaluators to minimize visual brand recognition.

To establish a standardized benchmark for accuracy, completeness, and clinical correctness during evaluation, expert ratings were anchored directly against current international and national clinical practice guidelines and consensus recommendations for MWA. Furthermore, our system has been validated and successfully implemented across several prior LLM evaluation studies by our group [16], [18], [19].

**Fig. 1:**
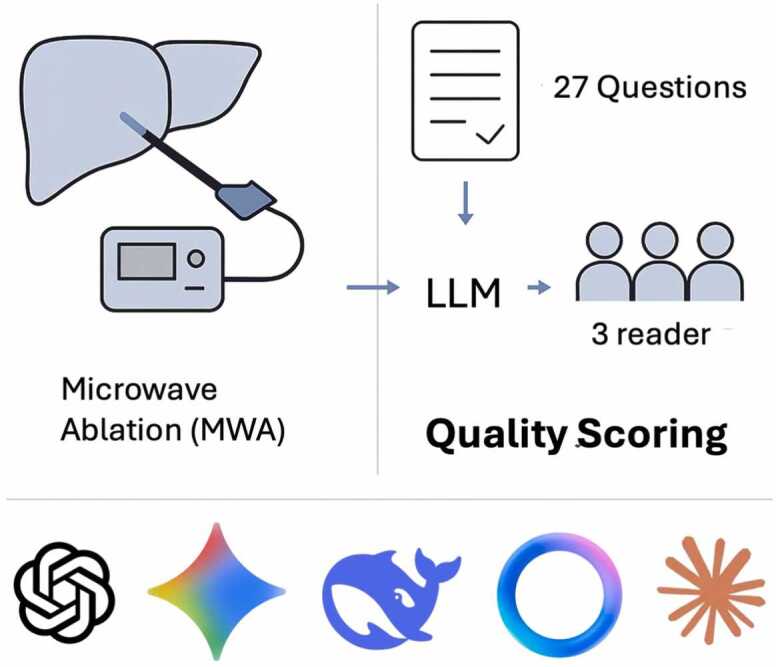
Schematic overview of the study design evaluating the performance of five large language models (LLMs)—ChatGPT, Gemini, Meta, Claude, and DeepSeek—in providing advice related to microwave ablation (MWA).

Fig. 1 shows the study design.

### 2.3 Scoring criteria

*I. Overall Quality Score (OQS)* 1 – Insufficient: incomplete, incorrect, or irrelevant answer 2 – Moderate: partially correct, missing essential points 3 – Good: accurate, covers most critical aspects 4 – Very Good: thorough, precise, and comprehensive.

*II. Understandability Score (US)* 1 – Insufficient: confusing or poorly structured 2 – Moderate: understandable but with issues in clarity or organization 3 – Good: clear and coherent with minor issues 4 – Very Good: highly clear, logical, and fluent.

*III. Implementability Score (IS)* 1 – Insufficient: impractical or vague 2 – Moderate: partially applicable but unrealistic in parts 3 – Good: mostly feasible with small adjustments needed 4 – Very Good: directly applicable in clinical workflow.

### 2.4 Statistical analysis

Data were summarized as mean ± standard deviation (SD), counts, or percentages. Descriptive statistics were used to outline LLM performance. Interrater reliability was assessed using a two-way mixed-effects intraclass correlation coefficient (ICC) model to evaluate the consistency among the three senior radiologists. ICC were classified as *moderate (0.41–0.60)*, *good (0.61–0.80)*, or *excellent (0.81–1.00)*.

Comparisons between the five LLMs were performed using the Kruskal–Wallis test. When significant differences were observed, post-hoc Mann–Whitney U tests were used to identify pairwise differences. To account for multiple comparisons and minimize Type I error, a Bonferroni correction was applied to all post-hoc pairwise tests. Statistical significance was defined as *p < 0.05*.

Additionally, rank epsilon-squared effect size estimates were calculated across all scoring criteria, indicating a large overall magnitude of difference (≥0.14) between the evaluated models.

All statistical analyses were conducted using IBM SPSS Statistics 28.0 (IBM, Armonk, NY, USA). Python 3.12 (Python Software Foundation) and large language models were used for chart generation and visualization.

## 3 Results

### 3.1 Overall findings

All five LLMs successfully responded to all 27 standardized questions regarding MWA. Each model produced clinically relevant answers, though notable differences in accuracy, clarity, and practical applicability were observed.

Across all evaluated criteria, ChatGPT achieved the highest performance, followed by Claude and DeepSeek**.** Gemini and Meta demonstrated lower scores, particularly in overall quality and implementability.

### 3.2 Interrater reliability

The interrater agreement was moderate to good, with ICCs ranging from 0.56 to 0.75. The highest agreement was observed for ChatGPT (ICC = 0.75), followed by Claude (ICC = 0.67)**,** DeepSeek (ICC = 0.57)**,** Meta (ICC = 0.59), and Gemini (ICC = 0.56). These values confirm a consistent and reproducible evaluation process among the three raters.

### 3.3 Overall model performance

When averaged across all questions and raters, ChatGPT achieved the highest Mean Quality Score (MQS) of 3.65 ± 0.54, outperforming Claude (3.47 ± 0.59) and DeepSeek (3.47 ± 0.60). Gemini (3.00 ± 0.55) and Meta (2.95 ± 0.61) scored significantly lower (p < 0.05).

Breaking down by scoring dimension:

• Overall Quality Score (OQS): ChatGPT achieved 3.58 ± 0.59, indicating superior accuracy and completeness compared with Claude (3.45 ± 0.61) and DeepSeek (3.30 ± 0.58).
• Understandability Score (US): ChatGPT again led with 3.77 ± 0.42, highlighting its clarity and coherent structure. DeepSeek (3.70 ± 0.53) and Claude (3.45 ± 0.57) followed closely.
• Implementability Score (IS): ChatGPT reached 3.59 ± 0.58, demonstrating high practical relevance of its responses. Claude (3.51 ± 0.59) and DeepSeek (3.44 ± 0.61) again performed well, while Gemini (2.87 ± 0.53) and Meta (2.85 ± 0.59) showed limited applicability.

**Fig. 2:**
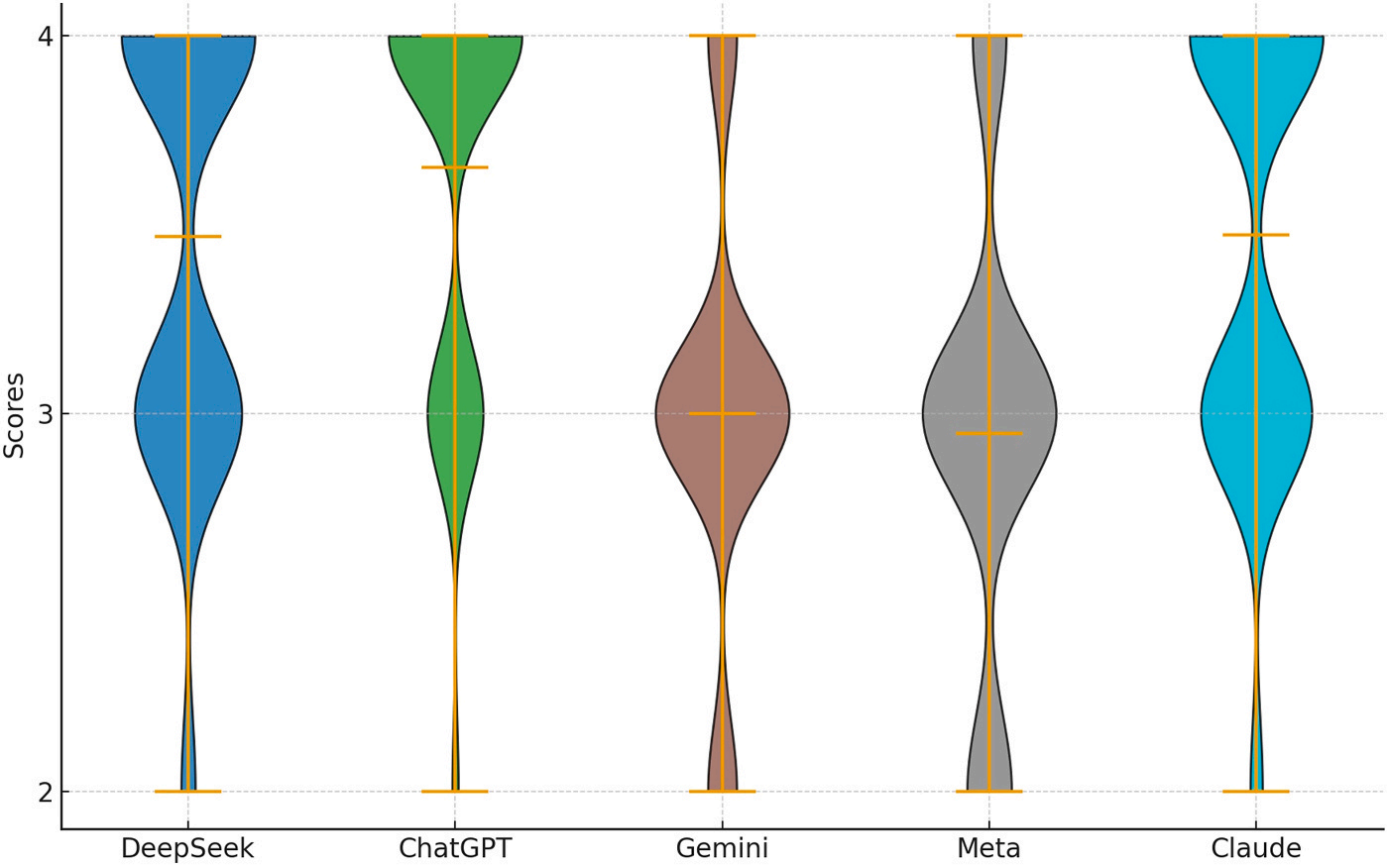
Violin plot visualizing the distribution of all 540 individual ratings across the evaluated large language models. Each violin represents the score distribution for one model, combining the Overall Quality, Understandability, and Implementability ratings. The plot highlights differences in score variability and median values between models.

Table 2 shows the summary of the overall model performance, while Fig. 2 shows the distribution of the ratings.

**Table 2:** Comparison of the performance of five large language models (LLMs)—ChatGPT, Gemini, Meta, Claude, and DeepSeek—across the three evaluation dimensions: Overall Quality Score (OQS), Understandability Score (US), and Implementability Score (IS). The Mean Quality Score (MQS) represents the average of all three metrics. Values are presented as mean ± standard deviation.

	ChatGPT	Gemini	Meta	Claude	DeepSeek
Overall Quality Score	3.58 ± 0.59	2.81 ± 0.45	2.75 ± 0.56	3.45 ± 0.61	3.3 ± 0.58
Understandability Score	3.77 ± 0.42	3.32 ± 0.52	3.26 ± 0.56	3.45 ± 0.57	3.7 ± 0.53
Implementability Score	3.59 ± 0.58	2.87 ± 0.53	2.85 ± 0.59	3.51 ± 0.59	3.44 ± 0.61
**Mean Quality Score**	**3.65 ± 0.54**	**3 ± 0.55**	**2.95 ± 0.61**	**3.47 ± 0.59**	**3.47 ± 0.6**

### 3.4 Subgroup performance

Performance varied slightly across the seven thematic subgroups (Table 3). ChatGPT achieved the highest MQS in most domains, with particularly strong results in *Effectiveness and Outcomes (3.73±0.44)*, *Risks and Complications (3.89±0.32)*, and *Post-Procedure Care (3.89±0.32)*.

**Table 3:** Mean Quality Scores (MQS ± SD) of five LLMs across seven thematic subgroups related to microwave ablation (MWA): Indications and Suitability, Procedure Details, Effectiveness and Outcomes, Risks and Complications, Post-Procedure Care, Patient Selection and Preparation, and Technical Aspects.

Subgroups	ChatGPT	Gemini	Meta	Claude	DeepSeek
**Indications and Suitability**	3.56 ± 0.5	3.17 ± 0.74	3.33 ± 0.63	3.5 ± 0.51	3.28 ± 0.74
**Procedure Details**	3.4 ± 0.65	2.96 ± 0.6	3 ± 0.64	3.51 ± 0.51	3.4 ± 0.65
**Effectiveness and Outcomes**	3.73 ± 0.44	2.89 ± 0.57	3.04 ± 0.37	3.67 ± 0.52	3.8 ± 0.41
**Risks and Complications**	3.89 ± 0.32	2.89 ± 0.58	2.75 ± 0.6	3.47 ± 0.48	3.61 ± 0.49
**Post-Procedure Care**	3.89 ± 0.32	3.19 ± 0.4	2.7 ± 0.54	3.22 ± 0.7	3.37 ± 0.49
**Patient Selection and Preparation**	3.74 ± 0.45	3.07 ± 0.39	2.89 ± 0.58	3.37 ± 0.63	3.33 ± 0.62
**Technical Aspects**	3.47 ± 0.7	2.94 ± 0.33	2.81 ± 0.67	3.22 ± 0.68	3.36 ± 0.59

Claude outperformed ChatGPT marginally in *Procedure Details (3.51±0.51)*, while DeepSeek achieved the best results in *Effectiveness and Outcomes (3.80±0.41)*. Gemini and Meta consistently underperformed across all subgroups.

These findings indicate that ChatGPT not only provides the most comprehensive and accurate answers overall but also maintains a consistent quality across different clinical categories relevant to MWA.

**Fig. 3:**
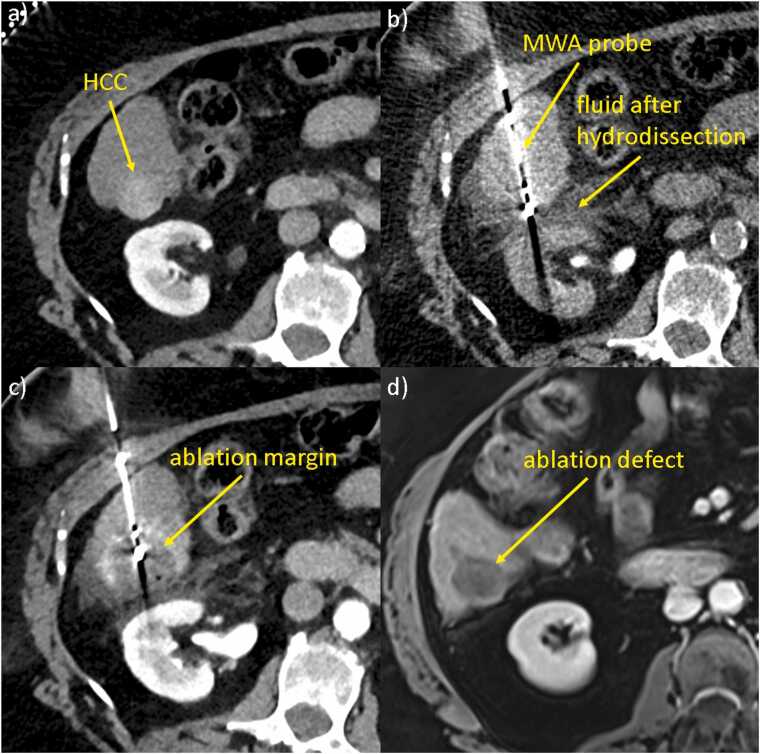
Case example of a successful MWA of an HCC in subcapsular location in segment 6. a) initial planning CT in arterial phase showing the hyperarterialized lesion. b) the MWA probe is precisely placed. A hydrodissection was performed to increase the distance to the right kidney. c) Immediately after the ablation a control CT scan is performed with the probe still in place, showing a complete ablation with a sufficient safety margin and without any complications. d) MRI control 4 weeks after showing the ablation defect without any evidence of rest tumor.

Fig. 3 shows an example of MWA.

## 4 Discussion

This study provides a comparative evaluation of five LLMs—ChatGPT, Claude, Gemini, Meta and DeepSeek—assessing their ability to provide advice on microwave ablation (MWA) using a score based on three key criteria: overall quality, understandability demonstrated by clarity and depth of understanding, and implementability showing the ease with which the information can be applied in a real world-scenario, each on a scale from 1 (lowest) to 4 (highest). This score has been successfully used in the evaluation of the performance of LLMs in other healthcare related topics [16], [18]. A score of 3 or more shows a valuable and dependable answer. All LLMs used in this study could provide useful answers in advising on MWA. However, the findings highlight significant differences in the performance of these models, with ChatGPT achieving the highest scores across all categories.

ChatGPT demonstrated superior performance with a mean score of 3.65, followed by Claude (3.47), Gemini (3.00), and Meta (2.95). ChatGPT’s strong performance across overall quality (3.58), understandability (3.77), and implementability (3.59) suggests its well-rounded capability in providing advice that is accurate, easily comprehensible, and practically actionable. The high understandability score of ChatGPT highlights its ability to deliver clear and concise responses, which is essential for supporting medical decision-making in specialized fields like MWA.

Claude and DeepSeek also performed well, with a mean score of 3.47 each.

When examining the individual subgroups, Claude performed slightly better than ChatGPT in the category *Procedure Details*, while DeepSeek performed somewhat better in *Effectiveness and Outcomes*. In the remaining categories, ChatGPT achieved the highest MQS. Notably, ChatGPT performed particularly well in the areas of *Effectiveness and Outcomes*, *Risks and Complications*, and *Patient Selection and Preparation* (all MQS > 3.7), whereas these same categories were relatively weaker for Meta and Gemini, which overall scored at a lower level.

The relatively close performance between ChatGPT, Claude and DeepSeek suggests, these models are viable options for medical applications, albeit with some differences in their strengths.

In contrast, Gemini (mean score 3) and Meta (mean score 2.95) exhibited lower performance, particularly in overall quality and implementability, both scoring below 3. These findings suggest that while Gemini and Meta have reasonable understandability scores (3.32 and 3.26, respectively), they are less adept at translating information into actionable advice. This limitation may stem from insufficient domain-specific training or algorithmic constraints that affect their ability to contextualize and prioritize relevant information.

Interestingly the ratings were a little bit lower than in a previously published study about the performance of ChatGPT on basic healthcare leadership and management questions [16], although the ChatGPT version used in this study was more advanced. The mean score for ChatGPT there was 3.75, compared to 3.65 in this study. The most likely reason is the more specialized topic in this study. However, the answers were rated by different readers in this study, so this might also contribute to differences in ratings.

A key consideration in evaluating LLM utility is the relationship between the three scoring dimensions: Overall Quality Score (OQS), Understandability (US), and Implementability (IS). Our analysis revealed a strong positive correlation between these metrics across the top-performing models. For instance, ChatGPT did not merely provide 'clear but inaccurate' advice; its high US was consistently matched by high OQS and IS scores, particularly in safety-critical domains such as 'Risks and Complications'. In contrast, lower-performing models like Gemini and Meta tended to score lower across all three dimensions simultaneously, suggesting that their primary limitation is a lack of domain-specific depth rather than a failure of communicative clarity. However, this association should be interpreted with caution. While high readability coincided with higher accuracy among top-performing models in our specific dataset, fluent and well-structured text can easily convey a false impression of clinical validity. In medical communication, an articulate presentation does not guarantee factual accuracy, and LLMs can generate highly plausible yet incorrect recommendations. Therefore, communicative clarity must never replace rigorous expert evaluation of underlying factual accuracy.

The interrater agreement ranged from 56% to 75% (best for ChatGPT), indicating moderate to good consistency between evaluators, comparable to two studies previously published using this score [16], [18]. While this level of agreement reflects some degree of subjectivity in the evaluation process, it also underscores the complexity of assessing LLM performance in a nuanced domain like MWA [13]. Future studies might improve reliability by involving a larger group of raters or by employing more objective scoring mechanisms, such as automated validation against standardized medical guidelines.

The differences in performance among the models have important implications for their potential integration into clinical practice. ChatGPT’s, DeepSeek’s and Claude’s high scores suggest they are more suitable for use in clinical decision support systems, where accuracy, clarity, and practical applicability are critical. However, the moderate interrater agreement, the need for domain-specific validation and the tendentially lower scores in more specialized categories such as technical aspects or procedure details emphasize that human oversight remains essential. Gemini and Meta, while less performant in this study, may benefit from targeted enhancements to improve their suitability for specialized medical applications.

A specific advantage of Gemini over the other LLMs was providing a reference, but this was not considered in the score used. It would certainly be desirable to include this feature in the other LLMs as well.

For the evaluation, we selected 27 questions distributed across seven distinct categories, each carefully designed to reflect real-world scenarios related to the use of microwave ablation. These scenarios addressed practical challenges, clinical decision-making situations, and management considerations that physicians frequently encounter in daily practice. With three to four questions per category, we aimed to capture and illustrate a wide range of relevant aspects of microwave ablation. Nonetheless, we recognize that certain areas may not have been fully represented in this study. Future research should therefore increase the number of questions.

LLMs have the potential to support both patients and referring physicians by providing timely, accessible, and comprehensible information about the benefits and limitations of microwave ablation. By generating tailored explanations, LLMs can help patients better understand the procedure, possible alternatives, and associated risks, thereby enhancing informed consent and shared decision-making. Similarly, referring physicians may benefit from rapid, evidence-based summaries that facilitate efficient communication with interventional radiologists and aid in selecting the most appropriate treatment strategy. Ultimately, the integration of LLMs into clinical workflows may contribute to more transparent discussions, reduced delays in decision-making, and improved patient-centered care. However, to the best of our knowledge, the applicability of LLMs in this context has not yet been investigated, and the performance of different LLMs in this regard has not been evaluated. In the context of MWA, LLMs have so far primarily been used as a tool for data extraction [20], [21]. However, Gordon et al. [22] could also show the potential beneficial use of LLMs in answering in general imaging-related questions. Chung et al. [23] and Schmidt et al. [24] could show the ability of LLMs in translating medical reports into a simplified summary understandable by patients. In this study, we were able to demonstrate that, through their ability to provide context-specific responses, LLMs offer an opportunity to alleviate the workload of radiology departments while delivering timely and helpful answers to both patients and referring physicians around the clock. In this respect, ChatGPT proved to be superior in our study, followed by DeepSeek and Claude. Nevertheless, the application of LLMs in this field must also take ethical and legal perspectives into account, particularly with regard to data security and liability [12]. Though, clear guidelines regarding the responsibility of healthcare professionals when using LLM chatbots are still lacking [25].

While high MQS indicate strong alignment with expert clinical expectations, these scores must not be equated with validated clinical correctness or autonomous diagnostic authority. However, the lack of a systematic citation ability and the models' inconsistent capacity to reference verifiable or peer-reviewed evidence remain a critical barrier to independent clinical use. Consequently, LLMs should currently be viewed as assistive tools for knowledge retrieval rather than primary decision-makers. Clinical risk management necessitates a "human-in-the-loop" framework, where LLM-generated insights are rigorously vetted by specialists to prevent the propagation of plausible but inaccurate medical advice.

This study also has some limitations: First, the evaluation used standardized, single-turn prompts that represent general clinical scenarios rather than complex, patient-specific cases or high-stakes edge cases. While this setup provided a controlled baseline for model benchmarking, it does not fully capture the nuanced decision-making required in challenging clinical situations where incorrect AI guidance could yield severe adverse consequences. Future evaluations should incorporate multi-layered case vignettes and high-risk edge cases to test the model's boundary conditions, provided that data privacy and regulatory standards are strictly maintained. Each LLM was evaluated using a single-turn, first-input approach. While this design mirrors real-world user interaction where decisions are made based on the initial output, it does not account for potential within-model response variance or stochasticity across multiple sessions. Because response consistency and reproducibility are critical prerequisites for clinical deployment, future studies should evaluate temperature settings, repeat-query stability, and longitudinal robustness of responses. The sources of information the LLMs used could not be checked. The current analysis treats ratings as independent observations. Future research should employ mixed-effects ordinal modeling to better account for the structural nesting of raters and questions within specific LLMs, providing more precise variance estimates. While expert consensus served as a surrogate for clinical truth, the OQS lacks an explicit "harm" category. Future evaluations should utilize granular rubrics that specifically penalize high-stakes errors, such as those related to microwave ablation. While clinical guidelines served as the underlying reference standard for expert evaluation, the scoring process relied on expert radiologist ratings without an automated, algorithmic guideline-benchmarking tool. A degree of subjectivity is therefore inherent to the evaluation system, as reflected by the moderate interrater agreement. Future studies should consider incorporating automated validation frameworks that benchmark model outputs directly against structured MWA guidelines in a standardized manner. Additionally, expert raters were not formally blinded to model identities. Although all model outputs were stripped of visual branding and converted to standardized plain-text formats to mitigate visual bias, complete anonymization remains difficult due to distinct model-specific “linguistic fingerprints”. Since the responses were presented to the raters in the same order each time, there is also a potential “fatigue bias.” However, as this study primarily focuses on comparing the different models, this “fatigue bias” is likely to have affected the rating of the respective models equally. Likewise, a certain degree of subjectivity is inherent to the scoring system used, as reflected by the only moderate interrater agreement. This study prioritizes p-values and Mean Quality Scores (MQS) to ensure direct comparability with the existing literature. However, the absence of formal effect size metrics (e.g., rank-biserial correlations) limits the interpretation of the magnitude of observed differences. Future research should employ mixed-effects ordinal modeling to better account for the structural nesting of raters and questions within specific LLMs, providing more precise variance estimates.

This study also raises broader questions about the role of LLMs in healthcare. Although their ability to deliver high-quality advice is promising, challenges remain, including ensuring consistent performance, mitigating risks associated with inaccurate or ambiguous recommendations, and addressing ethical and regulatory concerns [12].

Future research should focus on refining LLM training processes to improve their performance in niche areas like MWA. Incorporating domain-specific datasets, involving expert input during model development, and evaluating LLMs against standardized benchmarks could enhance their utility [13]. Expanding the scope of evaluation to include a broader range of medical scenarios and incorporating user feedback from healthcare professionals as well as patients will further improve their integration into clinical workflows.

## 5 Conclusion

All LLMs demonstrate strong potential as advising tools in MWA, with ChatGPT achieving the highest scores in this study. However, the observed variability across models and evaluators highlights the need for continued optimization, rigorous validation, and still careful oversight to ensure safe and effective use of LLMs in medical practice, especially if used in a patient-specific context.

## CRediT authorship contribution statement

**Sam Sedaghat:** Writing – review & editing, Writing – original draft, Visualization, Validation, Supervision, Software, Resources, Project administration, Methodology, Investigation, Formal analysis, Data curation, Conceptualization. **Hans-Ulrich Kauczor:** Writing – review & editing, Supervision. **Hyungseok Jang:** Writing – review & editing, Supervision, Methodology, Investigation, Formal analysis, Data curation. **René Mathy:** Writing – review & editing, Writing – original draft, Visualization, Validation, Software, Resources, Project administration, Methodology, Investigation, Formal analysis, Data curation, Conceptualization. **Fabian Schmitz:** Writing – review & editing, Methodology, Investigation, Formal analysis, Data curation.

## Consent for publication

Not applicable.

## Ethical approval

Not applicable.

## Studies human

All procedures were performed in compliance with relevant laws and institutional guidelines and have been approved by the appropriate institutional committee. Ethic approval was not applicable as the study did not include human subjects.

## Declaration of Generative AI and AI-assisted technologies in the writing process

Generative AI has been used for grammar correction, coding and chart generation.

## Funding

This research received no specific grant from any funding agency in the public, commercial, or not-for-profit sectors.

## Declaration of Competing Interest

The authors declare that they have no known competing financial interests or personal relationships that could have appeared to influence the work reported in this paper.

## Data Availability

Data available on request from the authors.
